# Peptides take centre stage in plant signalling

**DOI:** 10.1093/jxb/erv376

**Published:** 2015-08-05

**Authors:** Rüdiger Simon, Thomas Dresselhaus

**Affiliations:** Heinrich-Heine University Düsseldorf, Germany; University of Regensburg, Germany

Due to the presence of cell-wall material separating the surface of neighbouring cells, plants were expected to require many mobile ligands to exchange information among cells, tissues, organs, and with their environment. In particular, the classical plant hormones auxin, cytokinins, gibberellins, abscisic acid, and ethylene have long been considered to regulate growth and developmental processes as extracellular signalling molecules (Kende and Zeevart 1997). Until the 18 amino acid Systemin was discovered by Pearce *et al.* (1991) peptides were not known as regulators of signalling events in plants. The availability of the first plant genome in 2000, the generation of huge RNAseq data sets, and improved biochemical isolation procedures and gene prediction tools have shown that plants possess thousands of genes encoding putative secreted extracellular peptide ligands. These have now been distinguished into two major classes as cysteine-rich peptides (CRPs) with a length of about 40 to more than 100 amino acid residues and post-translationally modified small-peptides of about 10 amino acid residues (nonCRPs) (Matsubayashi, 2014). Notably, while nonCRPs regulate many intercellular communication processes during vegetative development and stress responses, CRPs are particularly abundant during plant reproduction. This special issue of the *Journal of Experimental Botany* now tries to provide a state-of-the-art insight into the numerous roles of some major plant peptide classes including CRPs as well as various nonCRPs such as CEPs, CLEs, IDAs, PEPs, PSKs, and others.

A recent report showed that hundreds of CRP genes are specifically regulated before and after fertilization in *Arabidopsis*. For example, 53% of the total number of female gametophyte-specific genes encode members of various CRP subclasses (Huang *et al.*, 2015). Starting with the gametophytic stage of plant reproduction, the first review in this special issue by Qu *et al.* (2015) describes how the pollen tube, the vehicle that transports the two immobile male gametes (sperm cells) through the sporophytic tissues towards the ovule for double fertilization, communicates with the surrounding tissues using a series of peptides and receptors from different classes. The underlying peptide signalling events, which are mainly mediated by secreted CRPs, serve also to avoid self- or alien fertilization as pre-zygotic hybridization barriers. They promote and guide the pollen tube to release its cargo to its ultimate targets, the egg and central cell, respectively. EMBRYO SAC1–4 (ES1–4) peptides belonging to the defensin subfamily of CRPs were previously shown to be required for sperm cell release. In this special issue, Woriedh *et al.* (2015) show that ES1–4 not only induce pollen tube burst during fertilization but also cause the swelling of fungal cells, the production of reactive oxygen species (ROS), and the burst of fungal cells at high concentrations. Interestingly, these apparently related effects on fungal hyphae cells and pollen tubes depend on different motifs within the ES peptides. Fertilization is then followed by a rapid loss of symplastic connections between the different compartments of the developing seed. From now onwards, apoplastic signalling is key to the control of developmental progression. With a focus on CRPs, Ingram and Gutierrez-Marcos (2015) discuss the diverse roles of peptides that control programmed cell death, mediate the communication between the developing embryo and endosperm with the maternal sporophytic seed tissues and, finally, how parental control of seed development is mediated by peptide signalling pathways.

Phytosulphokines (PSKs) belong to the post-translationally modified small-peptides and consist of only five amino acid residues. The disulphated peptide was initially described as a growth factor. Sauter (2015) reviews their synthesis, post-translational modifications, and perception by PSK receptors—plasma membrane-localized leucine-rich repeat receptor kinases (LRR-RLKs). Early in reproduction, PSK contributes to the funicular guidance of pollen tubes, but PSK has much wider roles in balancing the growth and defence responses. PSK receptors are regulated by Ca^2+^/calmodulin binding and may generate diverse signalling outputs via their protein kinase activity and cyclic GMP synthesis.

The IDA peptide is another nonCRP and was first described to promote organ abscission in *Arabidopsis*. Several IDA-like (IDL) peptides have now been analysed by Vie *et al.* (2015), and they found IDLs and the related PIP-peptides to be induced by biotic stresses. Both families are also related to CLE and CEP peptides, indicating their common evolutionary history. In cases where receptors have been unequivocally identified for these peptides, they belong to the LRR-RLK class suggesting also shared mechanisms for ligand-receptor interaction and the initiation of downstream signalling events. In an opinion paper, Butenko and Rüdiger Simon (2015) compare two signalling pathways that, at a first glance, appear to regulate very diverse processes in plant development. The CLV3 peptide belonging to the CLE family restricts stem cell number in shoot and floral meristems, while IDA peptides promote the abscission of lateral organs in the abscission zone. However, both peptides act through related RLKs and transcription factors, and Butenko and Simon argue that these pathways arose from a common ancient signalling system. Extending the analysis on CLE peptides, Czyzewicz, Shi *et al.* (2015) studied the roles of five CLE family members during lateral root initiation. Among these, CLE26 clearly affects root architecture and might modulate auxin transport or responses. CLE26 from *Arabidopsis* also reduced root size when applied to *Brachypodium*, indicating that CLE signalling pathways are evolutionary highly conserved. Legumes can form nodules as lateral root outgrowths that provide a nurturing environment for rhizobia, the symbiotic bacteria that deliver fixed nitrogen in return for carbohydrates. The carbon costs are high and nodule formation is tightly regulated to occur only under N-limiting conditions. Not surprisingly, plants regulate this expenditure via short and long range (between root and shoot) apoplastic signalling. Djordjevic *et al.* (2015) review the prominent roles of two root peptide families, CLE and CEP, and their receptors in N-demand signalling, lateral root formation, and the systemic auto-regulation of the nodulation pathway. Within the seed, embryos develop in close contact and synchrony with the surrounding endosperm. Xu *et al.* (2015) studied the role of CLE19, another CLE peptide which is expressed from early embryogenesis onwards in the protoderm and later in the L1 layer of the cotyledons, the vasculature, and the root tip. Using transgenic approaches involving antagonistic peptide technology, they identified CLE19 as a mobile signal that mediates the communication between embryo and endosperm in *Arabidopsis*. However, redundancies exist on both the peptide and the receptor side, setting the hurdles even higher for the identification of ‘linear’ signalling pathways and the assignment of a given peptide to a specific receptor and signalling outcome. Furthermore, loss-of-function alleles are either not available for many of the peptides described so far, due to the small size of their genes, or the lack of apparent phenotype(s) in a single mutant situation. Techniques for a more comprehensive functional analysis of peptides are therefore urgently needed, since novel peptides with signalling roles are being described at a rapid pace (see below). The generation of ‘antagonistic’ or dominant negative versions of a peptide that might block receptor function could provide one solution to this dilemma. Czyzewicz, Wildhagen *et al.* (2015) and colleagues from several different laboratories describe and critically discuss their attempts at creating such antagonistic peptide technology described above by exchanging individual amino acids in selected CLE and related IDA peptide′s primary sequences. They conclude that, although this technique has been successful in some cases (see also the article by Xu *et al*., this issue), it could greatly benefit from detailed structural information on the peptide–receptor interface. This is urgently required as the number of peptides is steadily increasing. Hastwell *et al.* (2015), for example, started to characterize 128 CLE peptide family members from soybean and common bean using the 32 *Arabidopsis* CLE genes for comparison and for separation into subgroups. CLE peptides from legumes have attracted (and deserve) considerable attention for their important role in the regulation of nodule formation (which is also addressed in the review by Djordievic *et al*. this issue). This work will provide a very useful reference source for future studies.

Other nonCRPs include PIPs, GLV/RGF/CLEL, and CEP peptides. In their review, Bartels and Boller (2015) take a fresh look at plant elicitor peptides, or PEPs. Elicitors signal the presence of microbes, damage, danger, or, very generally, stress situations. PEPs are found in many different plant species in contrast to Systemin, the first plant peptide involved in intracellular communication that is restricted to the Solanaceae (see above). Similar to PSKs, PEPs trigger Ca^2+^-release, cGMP production, and MAPK activation via the LRR-RLKs PEPR1 and 2. In addition to immune responses, the PEP systems now emerge as controlling root biomass and darkness-induced senescence. Lori *et al.* (2015) now extended the analysis of PEP peptides and their receptors, which, so far, were mostly studied in *Arabidopsis*, to other plant species and showed that PEPs are not recognized outside their family of origin. However, signalling events downstream of the PEP receptors, for example, ethylene production, are conserved, and PEPRs are interspecies compatible. Obviously, this finding opens the doors for approaches to re-engineer or exchange PEPRs between species to increase the immune system of crop plants. Klauser *et al.* (2015) then show that *PEP* expression is strongly induced by herbivores, and that PEPR mutant *Arabidopsis* plants are, consequently, more susceptible to the feeding attacks of *Spodoptera littoralis* (cotton leafworm). Lateral roots arise from the asymmetric divisions of xylem-pole pericycle cells, and this first cell division is affected by a surplus of GLV6, a peptide that is normally expressed in sites of lateral root initiation and the primary root. Fernandez and coworkers previously found that silencing of *GLV6* reduces both primary root length and lateral root density. GLV6 is a member of the GLV/RGF/CLEL family and several peptides of this group were found to promote root meristem growth by controlling PLT transcription factors through RLK dependent signalling pathways. Fernandez *et al.* (2015) now report that GLV6 affects nuclear migration which suggests that closely related peptides act through different mechanisms. As already discussed above, peptides are being cleaved from larger precursors and can undergo various post-translational modifications, so that the active form cannot be predicted from the primary sequence alone. Mohd-Radzman *et al.* (2015) now devised a novel peptide purification method from plant tissue and show here that the *Medicago truncatula CEP1* gene gives rise to five or more CEP peptides consisting of different domains with distinct hydroxylation and arabinosylation patterns. This work emphasizes again the importance of biochemical approaches: peptides must be isolated from the plant itself to define their different mature forms which can vastly affect their biological activities.

The number of new candidate signalling peptides is continuously increasing. Ghorbani *et al.* (2015) now describe their hunt for more and previously unknown short, secreted, peptide-encoding genes. Using comparative genomics, they searched through the genomes of 32 plant species and found both known and novel families. Combined with transcriptome data for lateral root development, they then focused on specific novel peptides for which they showed growth-altering effects after exogenous application. Environmental stressors, such as pathogens, drought or salinity, trigger physiological responses in the entire plant and a variety of plant hormones mediate local and systemic signalling. Chien *et al.* (2015) identified a novel salt-induced 11-amino-acid peptide in *Arabidopsis*, AtCAPE1, which is related to the cysteine-rich tomato immune regulator CAPE1. Surprisingly, they show that AtCAPE1 negatively regulates salt-tolerance and propose a role for this novel peptide in balancing immune and salinity responses. Plant lateral organ primordia are separated from the shoot meristem, their site of origin, by a specialized group of cells, which establish the boundary domain. Colling *et al.* (2015) now describe that, besides classical phytohormones, the novel cysteine-rich peptides TAXIMIN1 and 2 from *Arabidopsis* can affect organ separation. Both peptides are expressed in multiple tissues and it is likely that additional functions will be discovered upon further investigations. TAX peptides were first discovered in *Taxus baccata* and shown to modulate the biosynthesis of taxane, a desired anti-cancer drug. In *Arabidopsis*, TAX1 and 2 might indirectly affect organ separation by controlling the biosynthesis of a (unknown) hormone.

The majority of the peptides discussed here are secreted into the apoplast to control gene expression or function in adjacent cells, but the signalling cascades that they instigate are just single branches within larger gene regulatory networks. In the final paper of this special issue on peptide signalling in plants, Richards *et al.* (2015) use mathematical modelling to explore how a simple gene network, comprising the CLE40 peptide and the WOX5 transcription factor, can achieve stem cell homeostasis in the *Arabidopsis* root meristem. This paper points towards important future aspects of peptide signalling research to understand the cellular outputs of peptide–receptor interactions. In conclusion, this special issue of the *Journal of Experimental Botany* aims to provide an overview of some aspects of this exciting and innovative plant research field and gives some insights into the emerging technologies currently used to stimulate researchers to identify novel signalling peptides, their functions, receptors, and downstream signalling pathways. We hope you enjoy reading the various articles as much as we do.
